# A Preliminary Report of Real‐World Clinical Experience With Cold‐Crosslinked Hyaluronic Acid Fillers: A Roundtable and Case Series

**DOI:** 10.1111/jocd.71018

**Published:** 2026-07-20

**Authors:** Kristine Romine, Terrence Keaney, Jason D. Bloom, G. Jackie Yee, Heather Rogers, Ellen Gendler

**Affiliations:** ^1^ BelleSante Scottsdale Arizona USA; ^2^ SkinDC Dermatology Center Arlington Virginia USA; ^3^ Department of Dermatology George Washington Hospital Washington DC USA; ^4^ Division of Plastic Surgery, Department of Otorhinolaryngology Head & Neck Surgery, University of Pennsylvania Philadelphia Pennsylvania USA; ^5^ Bloom Facial Plastic Surgery Bryn Mawr Pennsylvania USA; ^6^ Plastic Surgery on Sunset Miami Florida USA; ^7^ Medspa on Sunset Miami Florida USA; ^8^ Department of Dermatology University of Washington Seattle WA USA; ^9^ Modern Dermatology Seattle WA USA; ^10^ Department of Dermatology NYU Langone Medical Center New York New York USA; ^11^ Gendler Dermatology New York New York USA

**Keywords:** clinical practice, cold‐crosslinking process, cold‐X technology, Evolysse Form, Evolysse Smooth, facial rejuvenation, hand rejuvenation, hyaluronic acid (HA) filler, real‐world use

## Abstract

**Background:**

Though data from clinical studies can inform real‐world application of new hyaluronic acid (HA) injectables, the eventual role of a given product in clinical practice is difficult to predict. Here, a panel of expert injectors share their experience with cold‐crosslinking process (Cold‐X Technology) HA fillers Evolysse Form (EVL_F_) and Evolysse Smooth (EVL_S_; Symatese, Chaponost, France).

**Methods:**

Twenty‐one expert dermatologists and facial plastic surgeons with an average of 24 years of experience (range 13–40 years) were provided with EVL_F_ and EVL_S_ to treat real‐world patients in their practices. All participants completed a survey on product use and overall experience and six participants participated in a 2‐h roundtable where case studies were discussed.

**Results:**

EVL_F_ was most often used in the cheeks (16/21), NLF (15/21), chin (13/21), and marionette lines (12/21), and EVL_S_ in the marionette lines (15/21), oral commissures (15/21), and lips (13/21), using a range of different injection techniques. Seventy‐six percent (16/21) of participants indicated that they were likely or very likely to incorporate EVL_F_ and EVL_S_ into their regular filler portfolio.

**Conclusions:**

Experience with EVL_S_ and EVL_F_ was positive: both are versatile, do not necessitate changes to existing workflows, and are able to achieve efficient outcomes in clinical practice.

## 1 Introduction

In 2024, over 5 million injections with hyaluronic acid (HA) fillers were carried out in the United States [1]. A wide range of HA filler products are available, each with a unique profile arising from differences in manufacturing. Features like product rheology (i.e., flow properties and lifting capacity), propensity to swell, extrusion force, and ease of degradation with hyaluronidase are measured objectively in a laboratory setting, while product safety, ability to reduce wrinkle severity, improve patient global aesthetic, and provide results satisfying to both patients and clinicians can be evaluated as part of clinical studies [2]. While this information informs clinical use and provides some picture of future use, the role that a given product will play in clinical practice, especially in the setting of a crowded marketplace, can be difficult to predict. This is in part because while most products are tested in single areas (e.g., nasolabial fold improvement), in clinical practice patients are injected in an “off label” manner in a wider range of areas, with different techniques, and in different planes than those tested directly in clinical trials. Furthermore, real‐world patients are more diverse than most clinical trial populations and generally judge results based on global improvement. Thus, in aesthetics, expert experience is highly valuable for providing information on possible patient niches, use and differentiators in real‐world practice, and special considerations.

Here, a panel of expert injectors was convened to discuss their experience using two recently FDA‐cleared products, Evolysse Form (EVL_F_) and Evolysse Smooth (EVL_S_; Symatese, Chaponost, France). Both of these fillers are produced using a unique cold‐crosslinking process (Cold‐X Technology), in which HA molecules are crosslinked into a gel under cold, rather than high‐heat conditions [3]. This reduces the amount of BDDE (1,4‐Butanediol diglycidyl ether) chemical crosslinker required and also preserves HA chain length: the final product includes longer chains, stabilized at a given rheologic parameters with less BDDE [3]. While clinical trials support both efficacy and safety in the NLF [3], there is a need to describe ways in which both EVL_F_ and EVL_S_ can be used in clinical practice and begin to develop an understanding of the niche that these products can fill or unmet needs they may be able to satisfy. Here, the authors share their early clinical experience with EVL_F_ and EVL_S_. This type of expertise is a first step toward understanding real‐world performance; however, these experiences must be supported by later real‐world studies and clinical data.

## 2 Methods

Between May 1st, 2025 and July 21, 2025, a panel of 18 expert dermatologists, facial plastic surgeons, plastic surgeon injectors, and 3 RN injectors with an average of 24 years of experience (range 10–40 years) were provided with EVL_F_ and EVL_S_ to use in the treatment of real‐world patients. Following an 8‐week period in which participants could treat patients, participants completed a survey on how the product was used and their individual experiences. Responses were collected and tabulated in a blinded fashion. Of these 21 clinicians, six submitted 2–3 case studies for discussion during a 2‐h roundtable, hosted by Premier Aesthetic Solutions LLC, and discussed their experience with EVL_F_ and EVL_S_ in more detail. For each of the patients treated, expert panelists provided the area treated, technique used, and number of syringes used. During the meeting itself, the discussion was focused on present and possible future real‐world use of both products and initial experience. Select case studies, information on product usage, and details of expert experience are presented here. All patients provided written consent for treatment and signed releases for publication and use of their photographs. The cases presented are not part of a formal study and therefore no institutional review board (IRB) approval was obtained; however, patients were treated in accordance with Good Clinical Practice and the principles outlined in the Declaration of Helsinki.

## 3 Results

### 3.1 Participant Experience

**FIGURE 1:**
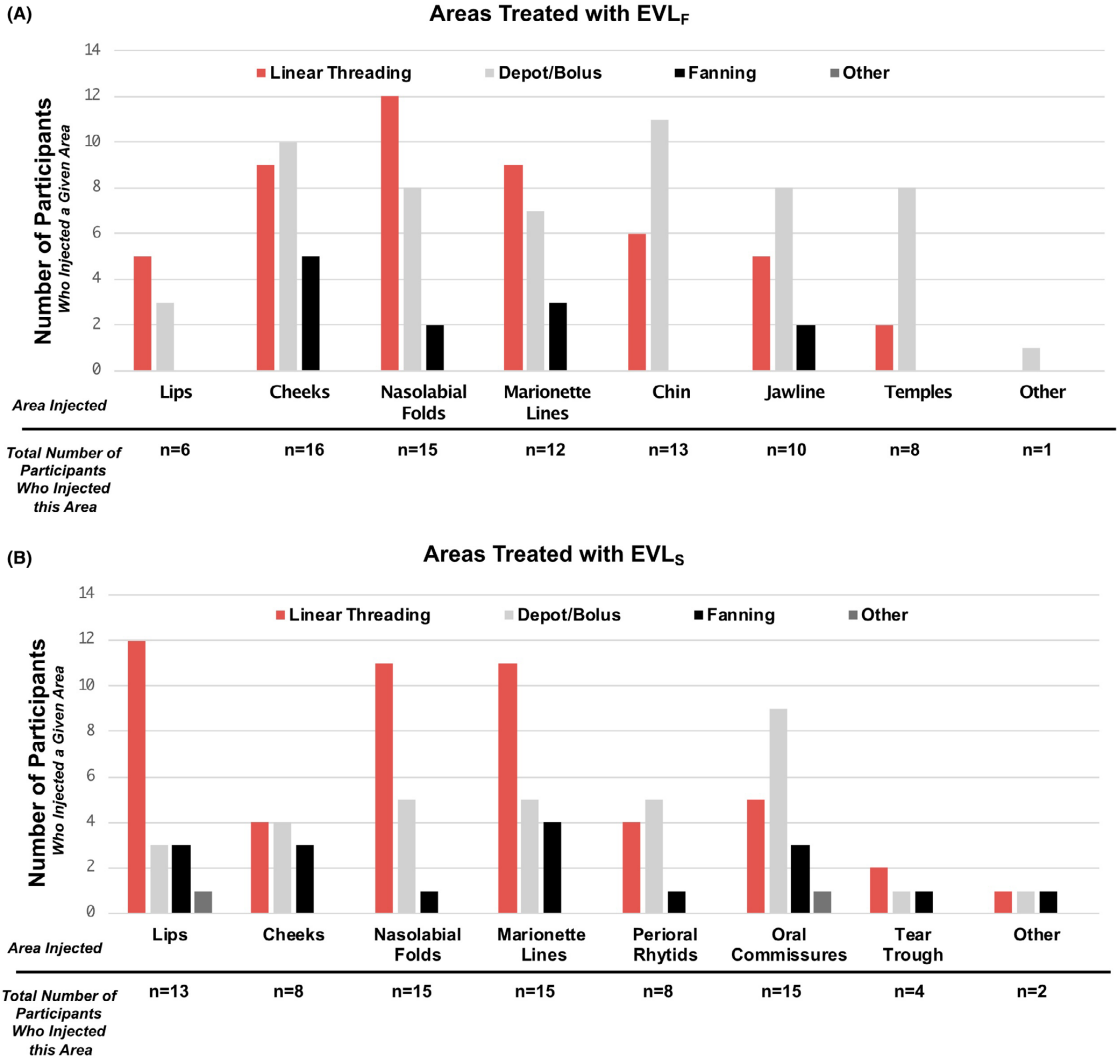
Areas treated at least once by survey participants with EVL_F_ (A) and EVL_S_ (B). The injection technique used in each area is shown (linear threading, depot/bolus, fanning, and “other”). The different areas injected are reflective of the distinct flow and lifting profile for each formulation.

Of the 21 user experience survey participants, most (65% [13/20]) treated at least 2 patients with EVL_F_ and 35% (7/20) treated ≥ 4 patients. Nearly all injectors used, on average, 1 (47% [9/19]) or 2 (47% [9/19]) syringes per patient. Panelists reported using a variety of injection techniques and treating a range of anatomical areas with EVL_F_ (Figure 1A), which was most often used in the cheeks (16/21), NLF (15/21), chin (13/21), and marionette lines (12/21).

For EVL_S_, 62% (13/21) participants treated at least two patients and 33% (7/21) treated ≥ 4 patients. On average, 46% (6/13) used 1 syringe, 31% (4/13) used 2 syringes, and 23% (3/13) used < 1 syringe per patient. Participants used EVL_S_ most often to treat marionette lines (15/21), the oral commissures (15/21), NLF (15/21), and lips (13/21; Figure 1B). During the roundtable, panelists clarified that in many cases, reports of injection of EVL_S_ in the cheeks and NLF occurred when there was some product remaining after lip or perioral injection.

**FIGURE 2:**
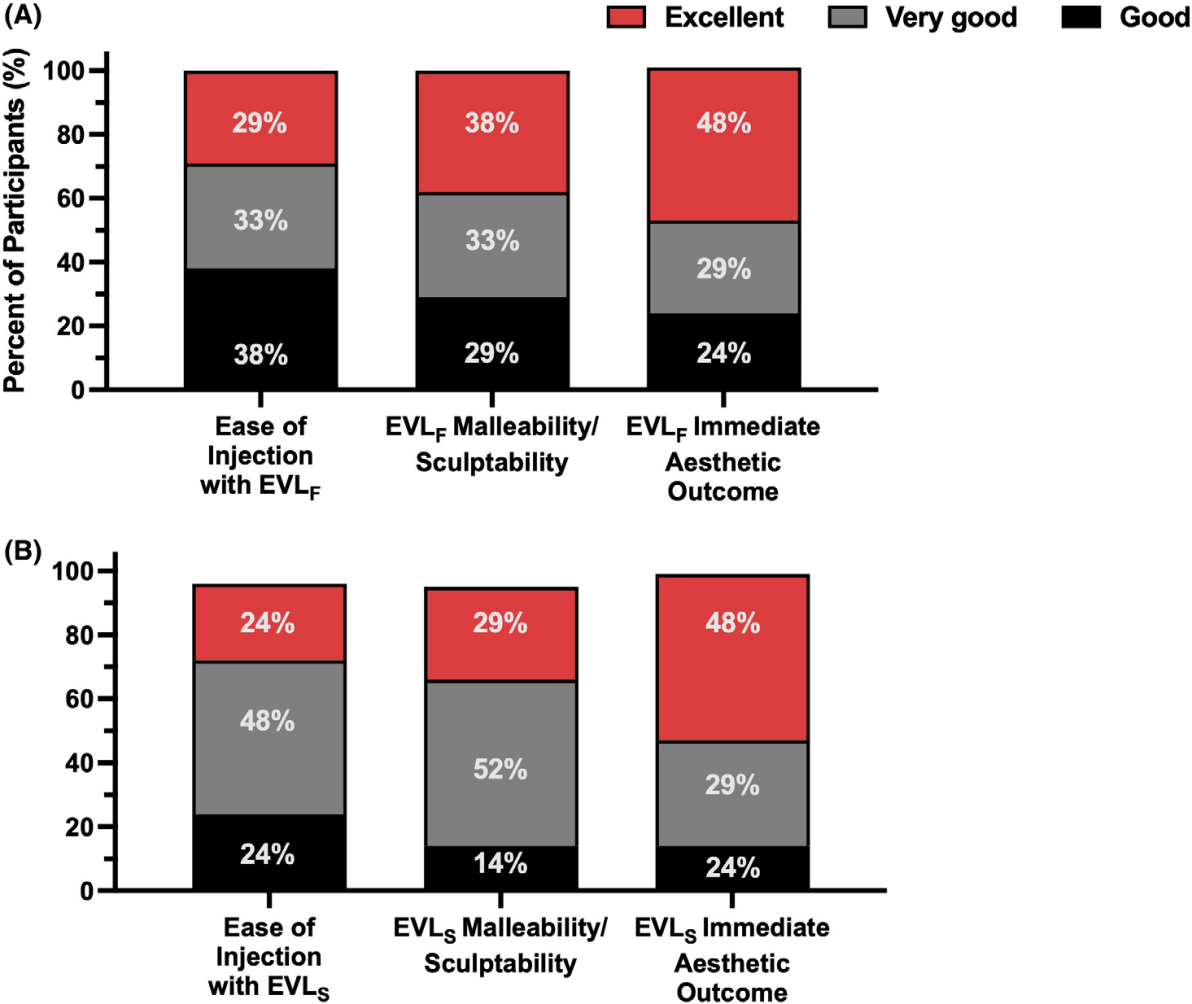
Participant user‐experience feedback for EVL_F_ (A) and EVL_S_ (B). Ease of injection, malleability, and immediate aesthetic outcome were rated positively by participants.

For both EVL_F_ and EVL_S_, panelists used a range of different injection techniques across different treatment areas. During the 2‐h round table, the six‐expert panel clarified that injection technique was guided by plane of injection and desired outcome, and did not need to be adjusted based on product behavior. A large majority of participants (81% [17/21]) agreed that both EVL_F_ and EVL_S_ are well suited for volume restoration and/or wrinkle treatment in post‐weight loss patients. For both products, ease of injection, malleability/sculptability, and immediate aesthetic outcome were highly rated (Figure 2) and 76% (16/21) indicated that they were likely or very likely to incorporate EVL_F_ and EVL_S_ into their regular filler portfolio.

### 3.2 Case Studies

**FIGURE 3:**
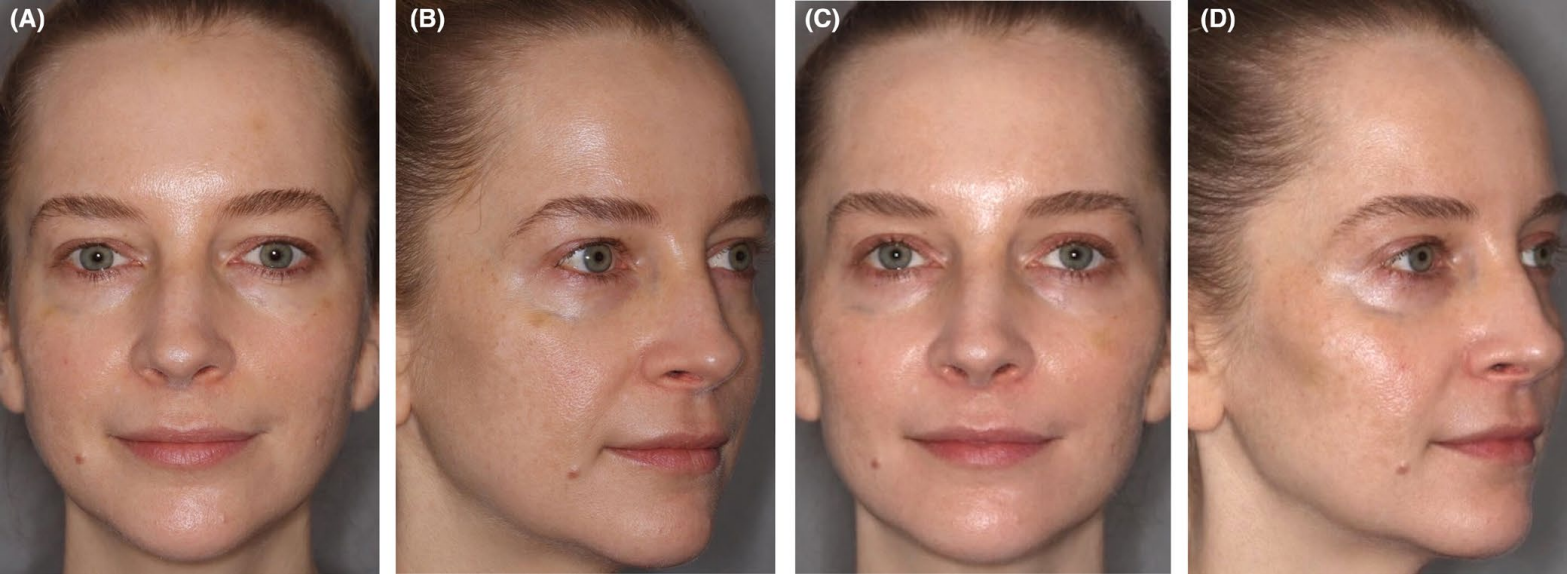
A 42‐year‐old female at baseline (A, B), and 2 weeks after (C, D) injection with 2 cc EVL_F_ to the cheeks and 2 cc EVL_s_ to the bilateral NLF and melomental folds.

**FIGURE 4:**
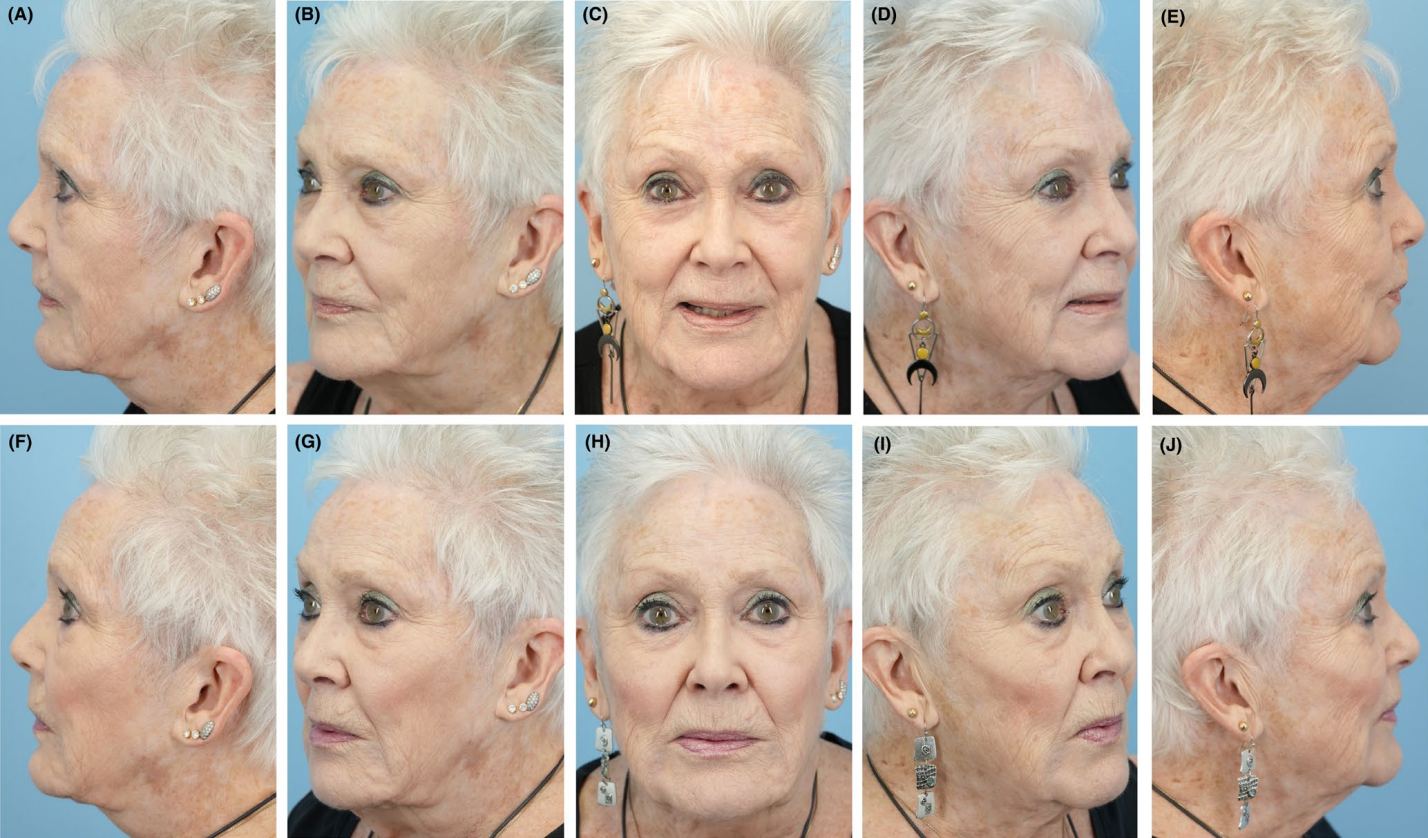
An 84‐year‐old female at baseline (A–E) and 4 weeks after (F–J) treatment with 2 cc of EVL_F_ in the midface (1 cc per side) and 1 cc of EVL_S_ in the marionette lines and pre‐jowl area.

**FIGURE 5:**
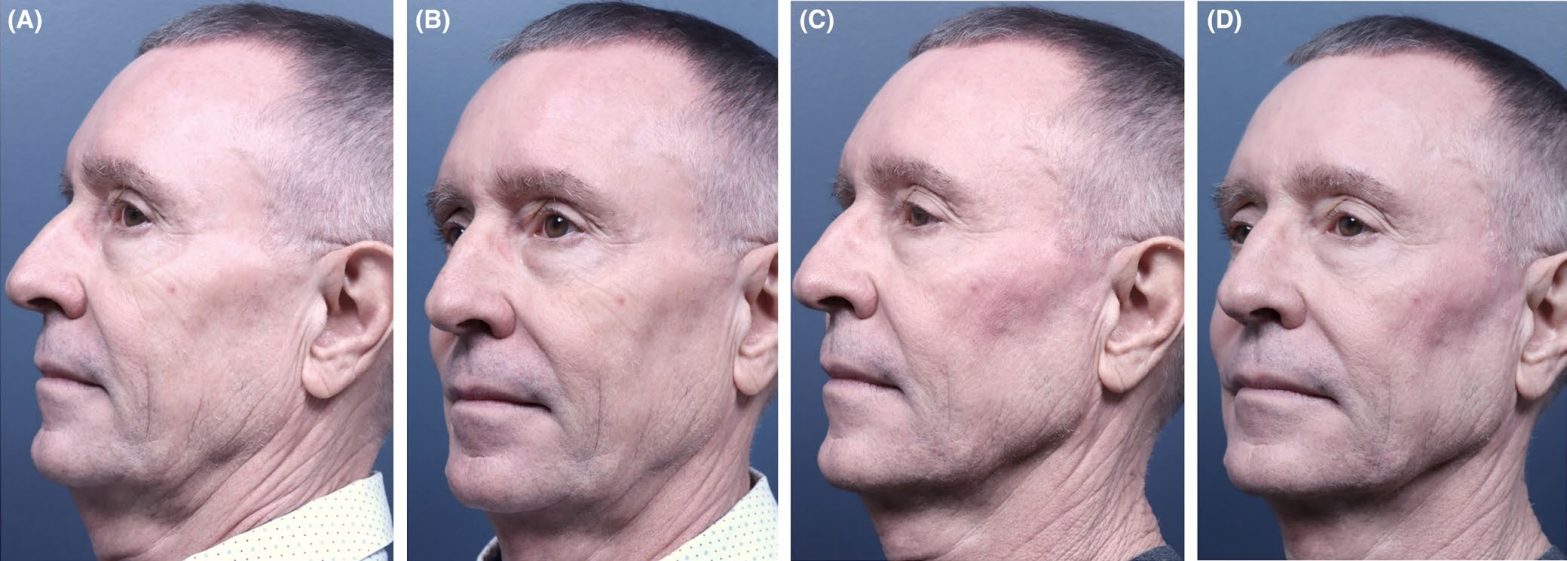
A 70‐year‐old male at baseline (A, B) and immediately after (C, D) injection with 2 cc of EVL_F_ in the jawline, marionette lines, pre‐jowl sulcus, and malar eminences (1 cc per side).

**FIGURE 6:**
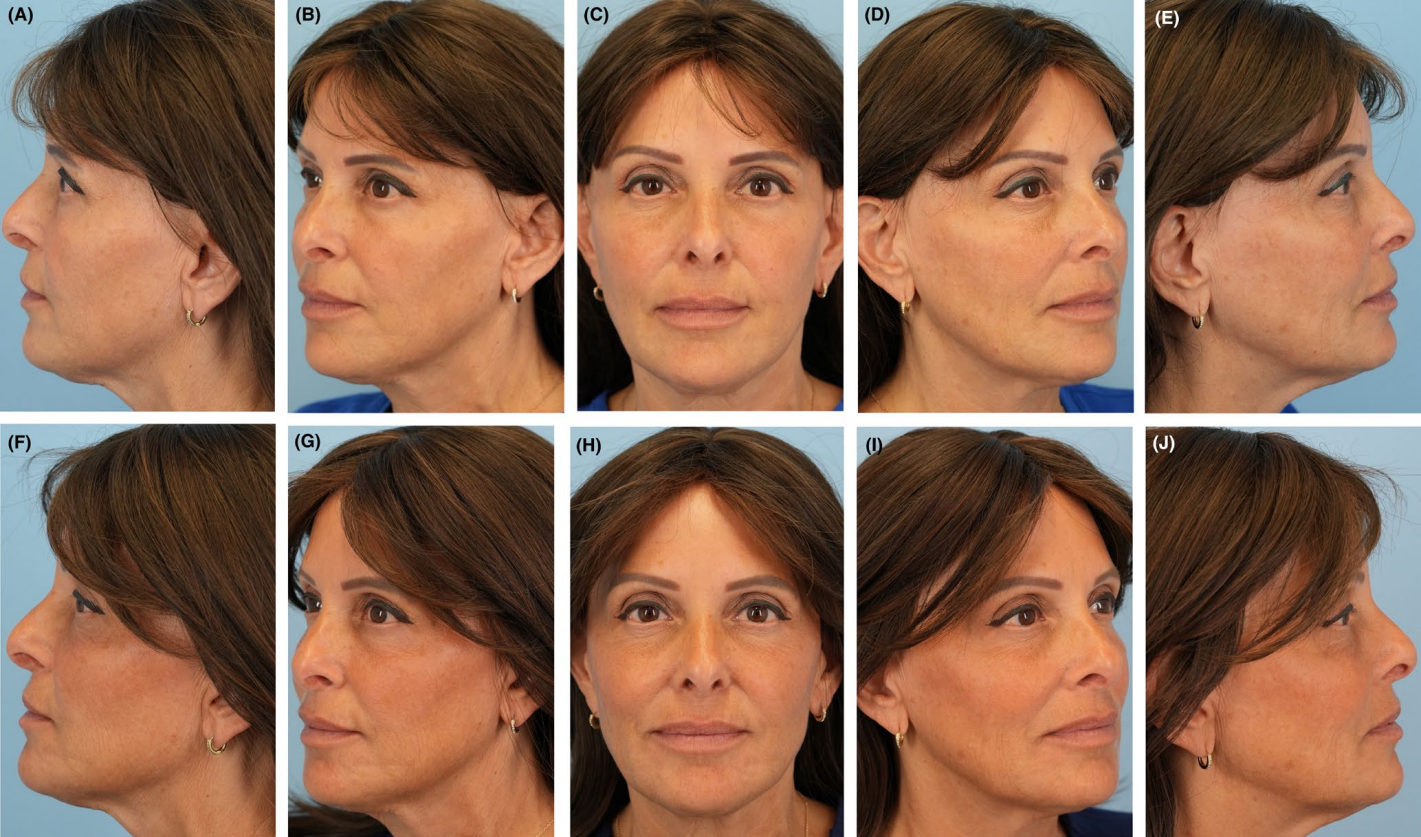
A 57‐year‐old female at baseline (A–E) and 2 weeks after (F–J) treatment with 1 cc of EVL_F_ to the midface (0.5 cc per side) and 2 cc of EVL_S_ to the marionette lines/oral commissure areas (1 cc per side).

**FIGURE 7:**
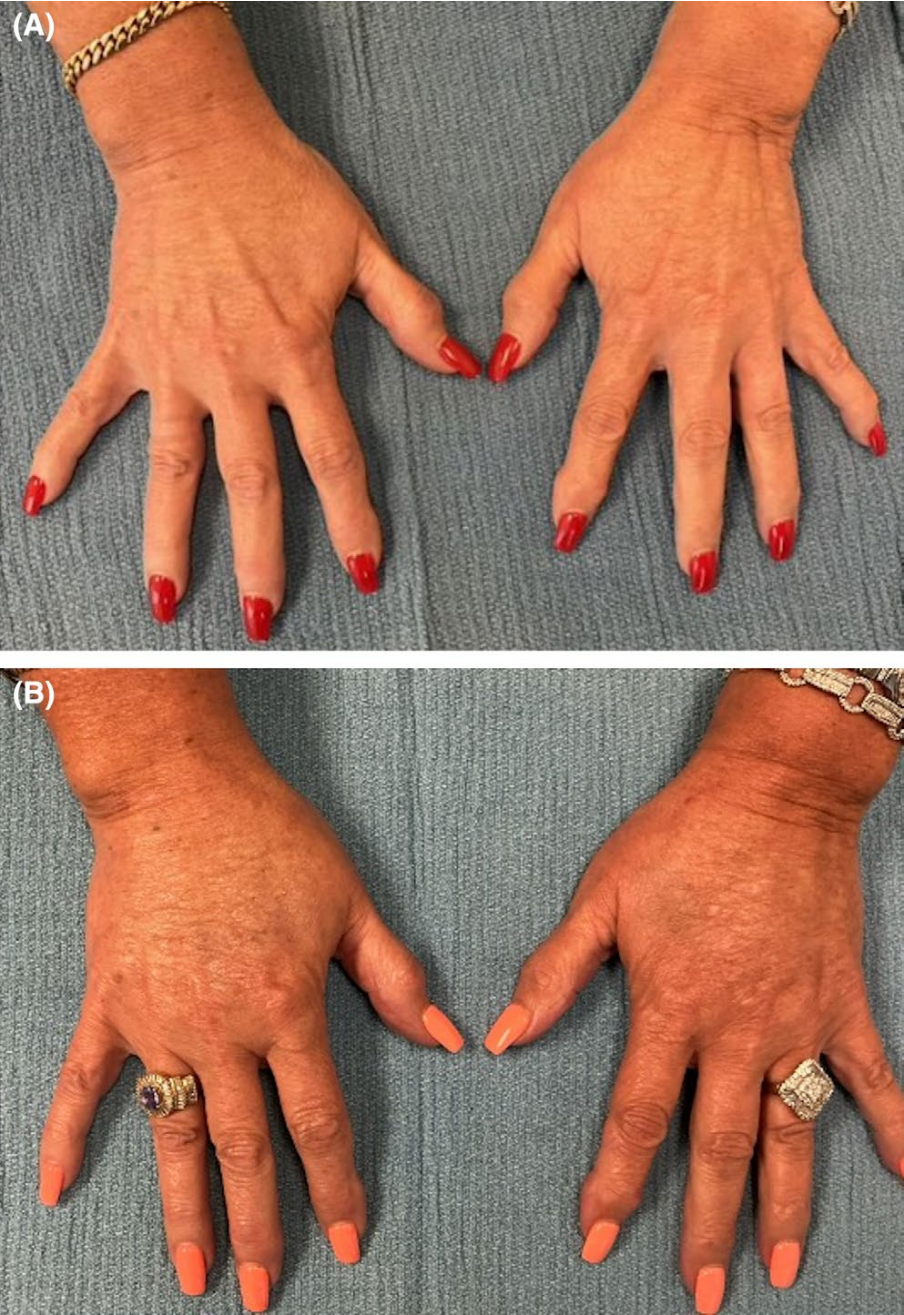
A 61‐year‐old female presented at baseline (A) and 2.5 weeks after treatment with 1 cc of EVL_F_ in the left hand and 1 cc of EVL_S_ in the right hand (B).

Cases from six patients discussed during the roundtable are shown in Figures 3, 4, 5, 6, 7. Patient and treatment information is provided. Product use is both on‐ and off‐label [4].

#### 3.2.1 Case 1

A 42‐year‐old female at baseline presented complaining of appearing hollow (Figure 3). The patient received 2 cc EVL_F_ to the bilateral cheeks delivered via cannula and 2 cc EVL_s_ to bilateral NLF and melomental folds delivered via 22G cannula. This patient is naturally thin and so treatment was applied with the intention of ensuring that her appearance is not too angular. This type of challenge is also often present in patients following massive weight loss. Following injection, there is soft, improved projection in the midface and diminished shadowing in the NLF and marionette folds.

#### 3.2.2 Case 2

An 84‐year‐old female presented desiring general facial rejuvenation (Figure 4). She had been treated many times before, about every 1.5–2 years with midface filler and marionette line filler, with the most recent treatment 1.5 years prior. Here, the patient was treated with 2 cc of EVL_F_ in the midface (1 cc per side). A total of 1 cc was placed on bone with a 27G needle and 1 cc in the superficial fat pads placed in a fanning pattern with a 25G, 1.5‐in. cannula. In addition, 1 cc of EVL_S_ in the marionette and pre‐jowl area was injected superficially with a 30G needle and fanned with a 25G 1.5‐in. cannula. Results included a pronounced change in midface laxity and projection as well as a dramatic reduction in shadowing in the marionette area. The apparent efficiency of EVL_S_ in the marionette fold is important because only a modest amount of filler can be used in this area in order to avoid making the jawline appear heavy.

#### 3.2.3 Case 3

A 70‐year‐old male presented for facial rejuvenation (Figure 5). He was treated with 2 cc of EVL_F_ in jawline, marionette lines, pre‐jowl sulcus, and malar eminences (1 cc per side). Improvement is particularly apparent in the marionette area and the mandibular border is better defined.

#### 3.2.4 Case 4

A 57‐year‐old female presented for facial rejuvenation. She had been treated many times prior with neuromodulators in the upper face about every 12 months and minimal filler to chin and marionette line areas. Her last filler injection was 6 months prior to the current treatment (Figure 6). The patient was treated with 1 cc of EVL_F_ to the midface (0.5 cc per side; 0.5 cc on bone with a 27‐gauge, ½‐inch needle, and 0.5 cc in the superficial fat pads with a 25‐gauge, 1.5‐in. cannula using fanning technique) and 2 cc of EVL_S_ (1 cc per side) to the marionette lines/oral commissure areas via superficial injection with a 30‐gauge needle and a 25‐gauge, 1.5‐in. cannula superficially using a fanning technique.

#### 3.2.5 Case 5

A 61‐year‐old female presented for hand rejuvenation (Figure 7). The was treated with 1 cc of EVL_F_ in the left hand and 1 cc of EVL_S_ in the right hand. Consistent with user experience data collected as a part of this event, both products were easily molded and spread.

## 4 Discussion

As new fillers are introduced into the market, practitioners are faced with the question of how to best integrate new technologies. For new HA fillers, which are introduced into a crowded marketplace, there is a need to carefully consider clinical need as well as any potential impact of uptake on workflow and business aspects of clinical care. This is complicated by the fact that many product differentiators presented at product launch are theoretical: for example, while nodules could theoretically be more common with more highly crosslinked fillers, clinically, the need for intervention most often arises from overfilling and/or product misplacement, rather than any differences in HA product composition. For EVL fillers, the presence of HA that is minimally processed and comprised of longer HA chains may theoretically have some advantage arising from appearing less foreign to the body; however, there is a need for data to support any claims of improved compatibility or safety. The expert experience collected here was intended to share with others the potential differentiators most relevant to injectors who already have a full suite of fillers in place. While expert experience is informative, later research in real‐world patients (including off label areas and injection techniques) is needed to characterize efficacy and durability in off‐label areas.

Here, survey participants used both EVL_F_ and EVL_S_ in a variety of patient types and a range of treatment areas. Overall, experience with both products was positive and neither necessitated significant adjustment to existing workflow: location/plane of injection, rather than the product properties, dictated injection methods used, a positive finding. Patient outcomes were positive and consistent with the positive performance of HA fillers in general and no new or unexpected adverse effects were reported. It is important to note; however, that no prospective data were collected. The versatility of EVL_F_ was noted as an advantage by several panelists. Of note, 83% of panelists see either EVL_F_ and EVL_S_ as a good fit for volume restoration or wrinkle treatment in post‐weight loss patients, a use consistent with device labeling [5]. Given the growing interest in mitigating the effects of GLP‐1‐mediated impact on facial aging, this is a relevant finding [6]. Indeed, several of the case study patients presented here are thinner patients.

Another challenge associated with integrating new injectable HAs into clinical practice is the fact that clinical studies leading to regulatory approval are carried out in a single area (e.g., NLF and cheeks), while in clinical practice, versatile fillers can be used in numerous areas of the face and injected into planes not studied in registrational trials. Here, EVL fillers were used in a range of areas commonly treated in real‐world clinical practice. Overall, participants believe that EVL_s_ is best suited for the treatment of lips, NLF, and marionette. For lip injections, panelists noted less swelling, faster recovery, and more persistent results that did not appear to fade as initial swelling dissipated, an important finding given the impact of the need for downtime on patient willingness to undergo treatment. Though this observation was independently reported by both injectors and patients, prospective data are needed, as swelling has been reported in the literature for lip injection [7]. Furthermore, the single comparative study showing a slightly lower rate of injection‐site swelling for EVL_S_ compared to RES_L_ (36% vs. 40%, respectively) was in the NLF, a fundamentally distinct anatomical area [6]. While complete rheology studies have not been published, European product IFUs report G′ values of 122 Pa for EVL_S_ [8]. For reference, Juvéderm Ultra Plus XC (Allergan, Irvine, CA) has a G′ of 148 Pa and Restylane Kysse (Galderma, Uppsala, Sweden), which has a G′ of 156 Pa [9]. Overall, in panelist opinion, EVL_F_ was best suited for treatment of the cheeks, temples, NLF, and chin. In areas where deeper placement and significant lift are required, EVL_F_ had enough firmness to achieved desired results. In the product IFU, a G′ values of 248 Pa is reported for EVL_F_ [10]. For comparison, Restylane Defyne (Galderma, Uppsala, Sweden) has a G′ of 260 Pa and Juvéderm Vollure XC/Volift (Allergan Aesthetics, Irvine, CA), which has a G′ of 273 Pa [9]. Overall, based on their experiences, 77% of panelists are likely (9/21, 43%) or very likely (7/21, 33%) to incorporate Smooth or Form into their regular filler portfolio.

Of note, EVL_S_ was noted as being particularly efficient, with a small volume achieving good results. In the registrational study, the mean volume injected for NLF correction was. 1.2 mL for EVL_F_ (range, 0.2–3.0 mL), 1.3 mL for RES_L_ (Restylane‐L [Galderma, Uppsala, Sweden]; range, 0.5–3.2 mL), and 1.0 mL for EVL_S_ (range, 0.4–1.9 mL), consistent with the clinical observations noted by panelists [3]. Importantly, the 1.0 mL volume for EVL_S_ was associated with noninferiority compared to RES_L_ , based on absolute difference in mean WSRS change from baseline to Month 6 (−0.22 [95% CI, −0.416 to −0.019]), favoring EVL_S_ . This potential improved efficiency is relevant for all patients, but could be a distinct advantage for weight loss patients, where substantial volume generally needs to be restored across multiple areas of the face.

The limitations of this manuscript are inherent. While the findings of this early real‐world experience roundtable are informative, they are not part of a prospective study. Thus, formal prospective metrics on safety, durability of effect, and patient satisfaction are lacking. This is particularly relevant for off‐label areas. Instead, the perspectives of experts in filler injection on a new set of products is shared. Additional quantitative data and clinical studies are needed to build upon the findings shared here.

## 5 Conclusion

Overall, experience with EVL_S_ and EVL_F_ as part of this 21‐clinician, real‐world treatment experience was positive. While mechanistic and theoretical differentiators between HA fillers can be difficult to prove or translate into real‐world performance, both EVL_S_ and EVL_F_ were versatile fillers, with EVL_S_ appearing to be particularly efficient. Outcomes in patients were positive and use of these EVL fillers did not necessitate changes to existing workflows.

## Author Contributions

K.R., T.K., J.D.B., G.J.Y., H.R., and E.G., each contributed case studies, participated in the survey, and participated in the roundtable. Ginny Vachon, listed in the acknowledgements, wrote the paper, and K.R., T.K., J.D.B., G.J.Y., H.R., and E.G., each revised and approved the final manuscript.

## Funding

The writing of this manuscript was supported by an arms‐length grant from Evolus. Evolus provided product for use in treating patients, but was otherwise not involved in data generation.

## Ethics Statement

The cases presented are not part of a formal study and therefore no institutional review board (IRB) approval was obtained; however, patients were treated in accordance with Good Clinical Practice and the principles outlined in the Declaration of Helsinki. All patients provided written consent for treatment and signed releases for publication and use of their photographs.

## Consent

All patients provided written consent for treatment and signed releases for publication and use of their photographs.

## Conflicts of Interest

Dr. Romine currently serves as a faculty member of the Evolus Academy and as a speaker and trainer for Galderma. She has served as an advisory board chair and on the Board of Directors for Evolus, as well as a speaker and trainer for Merz; Dr. Keaney serves as a Consultant, Advisory Board Member, Speaker's Bureau Member, Trainer, and Clinical Investigator for Merz and Allergan/AbbVie. He also serves as a Consultant and Advisory Board Member for Evolus and as an Investigator for Galderma and Medytox; Dr. Bloom serves as a Consultant, Advisory Board Member, Speaker's Bureau Member, Trainer, and Clinical Investigator for Galderma, Revance, and Allergan/AbbVie. He also serves as a Consultant and Advisory Board Member for Evolus; Dr. Yee serves as a consultant and trainer for Galderma and Revance. She also serves as a consultant and advisory board member for Evolus; Dr. Rogers has served as an Advisory Board Member for Evolus; Dr. Gendler has served as an Advisory Board Member for Evolus. Each of the above‐listed authors received product to use in their practice for this user‐experience program from the manufacturer and were reimbursed for their time and participation in the roundtable.

## Data Availability

Data are not publicly available.
